# TIMELESS promotes reprogramming of glucose metabolism in oral squamous cell carcinoma

**DOI:** 10.1186/s12967-023-04791-3

**Published:** 2024-01-04

**Authors:** Yafan Chen, Zhengyang Han, Le Zhang, Caihong Gao, Jingyi Wei, Xuyuan Yang, Yabing Han, Yunbo Li, Chunmei Zhang, Yixin Wei, Jiaqi Dong, Wenxing Xun, Weifu Sun, Taotao Zhang, Hui Zhang, Jingtao Chen, Peng Yuan

**Affiliations:** 1 Department of Nuclear Medicine, Tangdu Hospital, Air Force Medical University, 569 Xinsi Road, Xi’an, 710038 Shaanxi China; 2 Department of Clinical Laboratory, Sinopharm Dongfeng General Hospital, Hubei University of Medicine, Shiyan, 442008 Hubei China; 3 Department of Interventional Radiology and Pain Treatment, Tangdu Hospital, Air Force Medical University, Xi’an, 710038 Shaanxi China; 4 Xi’an Physical Education University, Xi’an, 710068 Shaanxi China; 5 The First Clinical Medical College, Shaanxi University of Chinese Medicine, Xianyang, 712046 Shaanxi China; 6 School of Nursing and Rehabilitation, Xi’an Medical University, Xi’an, 710021 Shaanxi China; 7 Medical College of Ankang University, Ankang, 725000 Shaanxi China; 8 The Second Clinical Medical School, Shaanxi University of Chinese Medicine, Xianyang, 712046 Shaanxi China; 9 Department of Stomatology, Tangdu Hospital, Air Force Medical University, 569 Xinsi Road, Xi’an, 710038 Shaanxi China; 10 Department of Ultrasound Diagnosis, Xi’an Children’s Hospital, 69 West Park Lane, Xi’an, 710002 Shaanxi China

**Keywords:** Oral squamous cell carcinoma (OSCC), *TIMELESS*, Glucose metabolism, *SIRT1*

## Abstract

**Background:**

Oral squamous cell carcinoma (OSCC), the predominant malignancy of the oral cavity, is characterized by high incidence and low survival rates. Emerging evidence suggests a link between circadian rhythm disruptions and cancer development. The circadian gene *TIMELESS,* known for its specific expression in various tumors, has not been extensively studied in the context of OSCC. This study aims to explore the influence of *TIMELESS* on OSCC, focusing on cell growth and metabolic alterations.

**Methods:**

We analyzed *TIMELESS* expression in OSCC using western blot, immunohistochemistry, qRT-PCR, and data from The Cancer Genome Atlas (TCGA) and the Cancer Cell Line Encyclopedia (CCLE). The role of *TIMELESS* in OSCC was examined through clone formation, MTS, cell cycle, and EdU assays, alongside subcutaneous tumor growth experiments in nude mice. We also assessed the metabolic impact of *TIMELESS* by measuring glucose uptake, lactate production, oxygen consumption, and medium pH, and investigated its effect on key metabolic proteins including silent information regulator 1 (*SIRT1*), hexokinase 2 (*HK2*), pyruvate kinase isozyme type M2 (*PKM2*), recombinant lactate dehydrogenase A (*LDHA*) and glucose transporter-1 (GLUT1).

**Results:**

Elevated *TIMELESS* expression in OSCC tissues and cell lines was observed, correlating with reduced patient survival. *TIMELESS* overexpression enhanced OSCC cell proliferation, increased glycolytic activity (glucose uptake and lactate production), and suppressed oxidative phosphorylation (evidenced by reduced oxygen consumption and altered pH levels). Conversely, *TIMELESS* knockdown inhibited these cellular and metabolic processes, an effect mirrored by manipulating *SIRT1* levels. Additionally, *SIRT1* was positively associated with *TIMELESS* expression. The expression of *SIRT1, HK2, PKM2, LDHA* and GLUT1 increased with the overexpression of *TIMELESS* levels and decreased with the knockdown of *TIMELESS*.

**Conclusion:**

*TIMELESS* exacerbates OSCC progression by modulating cellular proliferation and metabolic pathways, specifically by enhancing glycolysis and reducing oxidative phosphorylation, largely mediated through the *SIRT1* pathway. This highlights *TIMELESS* as a potential target for OSCC therapeutic strategies.

**Supplementary Information:**

The online version contains supplementary material available at 10.1186/s12967-023-04791-3.

## Introduction

Head and neck squamous cell carcinoma (HNSC), a significant global health concern, is most commonly and severely manifested as oral squamous cell carcinoma (OSCC) [1–3]. Alcohol, tobacco, betel, human papillomavirus, poor hygiene, and diet are the most known incidence of OSCC [3–5]. Presently, treatment options for OSCC predominantly include surgery, radiotherapy, chemotherapy, and immunotherapy. However, for patients suffering from recurrent, metastatic, and advanced stages of OSCC, treatment efficacy is notably poor, primarily involving radiotherapy and chemotherapy [6]. Furthermore, these treatment modalities are often associated with substantial side effects, complicating the management of OSCC patients [7]. Despite advancements in OSCC therapies, the overall prognosis for OSCC patients remains grim. Consequently, early diagnosis and the identification of critical molecular pathways in OSCC are imperative for enhancing patient prognosis and guiding treatment strategies.

Cancer development is a complex, multi-faceted process influenced by a network of genetic and metabolic factors. Intracellular metabolic disturbances, involving a variety of kinases, metabolic pathways, and epigenetic modulators, play a central role in the initiation and progression of cancer [8]. A hallmark of many cancers, including OSCC, is the upregulation of glycolysis, driven by the aberrant regulation of glycolytic enzymes and glucose metabolism pathways [9].

Circadian rhythms, inherent in most organisms, regulate an array of cellular, metabolic, physiological, and behavioral activities in mammals [10]. These rhythms are orchestrated at the molecular level by a series of core circadian genes, including *CLOCK, ARNTL, CRY1, TIMELESS, PER1,* and *NPAS2*, forming a transcriptional and translational feedback loop [11]. The link between disrupted circadian rhythms and cancer pathogenesis has emerged as a focal area of research. Studies have shown that anomalies in circadian rhythm can disrupt normal physiological processes, potentially leading to tumorigenesis and cancer progression [12]. For instance, diminished *PER1* expression in OSCC has been associated with advanced disease stages and decreased 5-year survival rates [13, 14]. *PER1*’s role extends to influencing cellular metabolic pathways, such as glycolysis, via the PI3K/AKT signaling pathway, thereby impacting OSCC development and progression [15].

Several studies have highlighted the prominent expression of the *TIMELESS* gene in various cancer types. Specifically, high expression of *TIMELESS* has been observed in human breast cancer tissues, with two associated SNPs (rs2291738 and rs7302060) linked to an increased risk of breast cancer [16]. Functional studies further reveal that reducing *TIMELESS* levels significantly curtails the proliferation of the breast cancer cell line MCF-7 [17]. Similarly, in cervical cancer, *TIMELESS* overexpression is associated with a higher risk of recurrence and poorer recurrence-free survival rates, suggesting its potential as an independent prognostic marker in the early stages of this cancer [18]. Moreover, *TIMELESS* has been implicated in the development and progression of various other cancers, including nasopharyngeal carcinoma, prostate cancer, lung cancer, colorectal cancer, and kidney cancer [19–23]. In line with these findings, our preliminary studies indicate a specific upregulation of *TIMELESS* in OSCC tissues, correlated with a negative impact on prognosis. The objective of our current research is to explore the role of *TIMELESS* in OSCC. We aim to delineate the relationship between *TIMELESS* expression and OSCC development, thereby understanding its potential influence on the progression of this malignancy.

## Materials and methods

### Public data and clinical samples collection

We utilized the TCGA and the CCLE databases to study the expression patterns of core circadian genes in head and HNSC patients and corresponding cell lines. In addition, with ethical clearance from the Air Force Medical University's Ethics Committee, we systematically collected 133 tissue samples of OSCC and their adjacent noncancerous tissues. These samples were obtained from patients treated at the Second Affiliated Hospital of Air Force Medical University between January 2021 and December 2022. Informed consent was obtained from all patients before sample collection. Then we analyzed the relationship between the expression of *TIMELESS* and the prognosis of OSCC patients by collecting the prognosis data for all patients.

### Cell lines and culture and transfections

The OSCC cell lines SCC-4, SCC-9, SCC-15, SCC-25, CAL-27, and the normal oral cell line NOK were acquired from the Dental Hospital of Air Force Medical University. These cell lines were cultured in DMEM (Procell, PM150210, China) or DMEM/F12 (Procell, PM150312, China), supplemented with 1% penicillin/streptomycin (Procell, PB180120, China) and 10% fetal bovine serum (FBS) (Procell, 164210, China). The cultures were maintained at 37 °C in a humidified atmosphere containing 5% CO2. For specific cell lines (SCC-4, SCC-15, SCC-25, and SCC-9), hydrocortisone (400 ng/mL, Procell, CM-0571, China) was added to the medium.

To assess the role of *TIMELESS*, SCC-15 cells were infected with a *TIMELESS* overexpression lentivirus, while SCC-9 cells were transfected with a *TIMELESS* knockdown lentivirus. Corresponding control groups were also established for comparative analysis. The lentiviruses were developed by Shanghai OBiO Biotechnology Co. Ltd., and the *SIRT1* overexpression vector was created by Gene Pharma (Shanghai, China). We utilized the GL401 vector (pcSLenti-U6-shRNA-CMV-puro-WPRE) for generating *TIMELESS* shRNA lentivirus and control lentivirus, and the GL186 vector (pcSLenti-CMV-MCS-3xflag-PGK-Puro-WPRE) for the *TIMELESS* overexpression lentivirus and its empty vector control. Transfection procedures were carried out according to the manufacturer’s instructions. Post-transfection, cells stably expressing the desired genes were selected using puromycin (Beyotime, China) for five days. The transfection efficiency was verified using quantitative real-time PCR (qPCR) and Western blot analysis.

### Quantitative real time-PCR (qRT-PCR) analysis

Total RNA was extracted from tumor tissues and cells using Trizol reagent (Thermo Fisher, 16096020, USA). The extracted RNA was then reverse transcribed into cDNA using a kit from Takara (RR047A, Japan). Subsequent real-time PCR analysis was conducted using a Takara kit (DRR081, Japan). Primers for the genes of interest were synthesized by Sangon Biotech (Shanghai, China), and β-actin was utilized as an internal reference gene for normalization. The primer sequences used were as follows:

- TIMELESS forward primer: 5′-TCTGATCCGCTATTTGAGGCA-3′, reverse primer: 5′-GGCAGAAGGTCGCTCTGTAG-3′.
- SIRT1 forward primer: 5′-GTCACACTTAGCACAGAGCAGC-3′, reverse primer: 5′-TTTCTCCAGTACATACACAAC-3′.
- HK2 forward primer: 5′-GAGCCACCACTCACCCTACT-3′, reverse primer: 5′-CCAGGCATTCGGCAATGTG-3′.
- PKM2 forward primer: 5′-AAGGGTGTGAACCTTCCTGG-3′, reverse primer: 5′-GCTCGACCCCAAACTTCAGA-3′.
- LDHA forward primer: 5′-TATCTAATGAAGGACTTGGCGGATGAG-3′, reverse primer: 5′-GGAGTTCGCAGTTACACAGTAGTC-3′.
- GLUT1 forward primer: 5′-TTGCAGGCTTCTCCAACTGGAC-3′, reverse primer: 5′-CAGAACCAGGAGCACAGTGAAG-3′.
- β-actin forward primer:5′-CCCAGCCATGTACGTTGCTA-3′, reverse primer: 5′-TCACCGGAGTCTCACGAT-3′.

Each experiment was replicated three times. The relative expression levels of the target genes were calculated using the 2^−ΔΔCt^ method.

### Hematoxylin and Eosin (HE) and Immunohistochemistry (IHC) Staining

For histological examination, all collected tissue specimens underwent formalin fixation and were embedded in paraffin. Hematoxylin and eosin (HE) staining was performed using the Solarbio kit (G1120, China) to assess the general tissue morphology. For immunohistochemical analysis, we adhered to the established staining protocols as outlined in previous literature [24] and the manufacturer's instructions provided by MXB (KIT-9730, China).

The processed tissue sections were first deparaffinized and rehydrated. Antigen retrieval was then performed using a citrate-based solution. Following this, sections were incubated in a sequential manner with appropriate primary and secondary antibodies. The visualization of the target antigens was achieved using diaminobenzidine (DAB) as the chromogen, followed by counterstaining with hematoxylin to highlight the nuclei. Quantification of immunostaining was based on two key criteria: the proportion of positively stained cells and the intensity of staining. The proportion of positive cells was scored on a scale from 0 to 4: 0 for less than 10% positive cells, 1 for 10–25%, 2 for 26–50%, 3 for 51–75%, and 4 for over 75% positive cells. Staining intensity was graded as follows: 0 indicating no staining, 1 for weak staining, 2 for moderate staining, and 3 for strong staining. A composite staining score, ranging from 0 to 12, was then calculated by multiplying the intensity score by the proportion score. For the immunohistochemistry assays, we used the Ki67 antibody (AF0198) from Affinity Biosciences, and the PCNA antibody (60097-1-Ig), SIRT1 antibody (13161-1-AP), HK2 antibody (66974-1-Ig), PKM2 antibody (60268-1-Ig), GLUT1 antibody (66290-1-Ig), LDHA antibody (66287-1-Ig), and TIMELESS antibody (14421-1-AP) from Proteintech Group, Inc.

### Western blot analysis

Cellular proteins were isolated using RIPA lysis buffer (Beyotime, P0013B, China), supplemented with phenylmethylsulfonyl fluoride (PMSF, Beyotime, ST506, China) for protease inhibition. The total protein content was quantified using the BCA Protein Assay Kit (Beyotime, P0012S, China). These proteins were then resolved by SDS-PAGE and electrotransferred onto PVDF membranes, with the transfer tailored to the molecular weights of the proteins of interest. β-actin served as an internal reference for protein loading and transfer efficiency.

Following the transfer, the membranes were blocked with 5% non-fat milk in TBST to minimize non-specific antibody binding. They were then incubated first with primary antibodies and subsequently with secondary antibodies. The primary antibodies used were specific for β-actin (66009–1-Ig), Cyclin D1 (60186–1-Ig), Cyclin E1 (11554–1-AP), CDK2 (10122–1-AP), CDK4 (11026–1-AP), all sourced from Proteintech Group, Inc. For secondary detection, Goat Anti-Mouse IgG (CW0102) and Goat Anti-Rabbit IgG (CW0103) were obtained from Jiangsu Cowin Biotech Co., Ltd.

Protein bands were visualized using the ECL luminescent reagent (Beyotime, P0018FS, China). The Image J software was employed for the development and quantitative analysis of the bands, specifically assessing their gray value intensities. This process enabled an accurate determination of the relative expression levels of the targeted proteins in the samples.

### MTS cell viability assay

For the MTS assay, we seeded (0.5–1) × 10^4^ cells per well in a 96-well microplate, each well containing 100 μL of culture medium, and incubated them under standard conditions. After 24 h, 10 μL of MTS solution (Bestbio, BB-4204, China) was added to each well. The plates were then incubated at 37 °C for a duration ranging from 1 to 4 h. Cell viability was determined by measuring the absorbance at 490 nm, representing the optical density (OD) of each well.

### Clonal formation experiment

In the clonal formation assay, we plated 1000 cells in each well of 6-well plates. These cells were cultured for approximately two weeks, after which they were stained with crystal violet (Beyotime, C0121, China) to visualize and count the colonies. Each experimental condition was replicated in three separate wells to ensure consistency and reliability of the results.

### 5-Ethynyl-2′-deoxyuridine(EdU)assay

For the EdU assay, a cell density of 4 × 10^3^ to 1 × 10^5^ cells per well was maintained in 96-well plates. The cells were treated with 50 μM EdU (Ribobio, C10310-1, China) for 2 h, then fixed and stained using the Apollo and Hoechst solutions. The percentage of EdU-positive cells, indicating active DNA synthesis, was assessed under a live-cell imaging system.

### Cell cycle analysis

To analyze the cell cycle, (5–10) × 10^6^ cells were harvested, washed twice with cold PBS, and fixed in 70–90% ethanol at − 20 °C overnight. Post-fixation, cells were washed and resuspended in PBS, treated with 20 μL of RNase A for 30 min at 37 °C, and stained with 400 μL of propidium iodide (PI) solution (Bestbio, BB-4101, China) for 30 min in the dark at 4 °C. The stained cells were then subjected to flow cytometric analysis to determine the distribution of cells across different phases of the cell cycle.

### Measurement of glucose uptake, lactate production, pH, and oxygen consumption rate

Cells were seeded in 96-well plates at a density of 2000 cells per well. After washing thrice with PBS, glucose uptake was assessed using the Glucose Uptake Colorimetric Assay Kit (Sigma, MAK083, USA). Lactate secretion in the cell culture medium was quantified using the Lactate Assay Kit (Sigma, MAK065, USA). Both assays' results were normalized to the total protein content of each sample. The pH of the medium was measured with a pH meter (Bohlertech Technology, China), and the oxygen consumption rate (OCR) of the cells was determined using the Hansatech Oxytherm system (Hansatech, UK). These experiments were performed in triplicate and replicated three times to ensure consistency.

### Subcutaneous tumor xenografts in nude mice

Male nude mice (BALB/c), aged 4–6 weeks, were acquired from the Experimental Animal Center of the Air Force Medical University. These mice were housed in a specific pathogen-free facility and randomly divided into two groups, each comprising five mice. Following a week of acclimatization, SCC-9 cells, either with *TIMELESS* knockdown or control cells (1 × 10^7^), suspended in diluted Matrigel Matrix (BD, 354234, USA), were subcutaneously injected into the flanks of the mice. The growth of the tumors was monitored every five days, with tumor volume measurements recorded. Tumor volume was calculated using the formula: width^2^ × length × 0.5. The health and mortality of the mice were monitored throughout the experiment. At the study's conclusion, tumors were excised, weighed, photographed, and processed for formalin fixation and paraffin embedding. This experimental protocol was approved by the Ethics Committee of the Air Force Medical University.

### Statistical analysis

The analysis of all collected data was conducted using GraphPad Prism version 9.0 software (GraphPad Software Inc., La Jolla, CA, USA). Results were presented as mean values ± standard error of the mean (SEM). For comparisons within the same group, the paired t-test was employed, while the unpaired t-test was utilized for analyzing differences between two separate groups. In instances involving more than two groups, a one-way analysis of variance (ANOVA) was applied. For survival analyses, Log-rank test was used to analyze the survival curve. Correlation analysis was performed using Pearson’s correlation. A p-value of less than 0.05 was set as the threshold for statistical significance.

## Results

### Upregulation of TIMELESS in OSCC and its correlation with poor prognosis

**Fig. 1:**
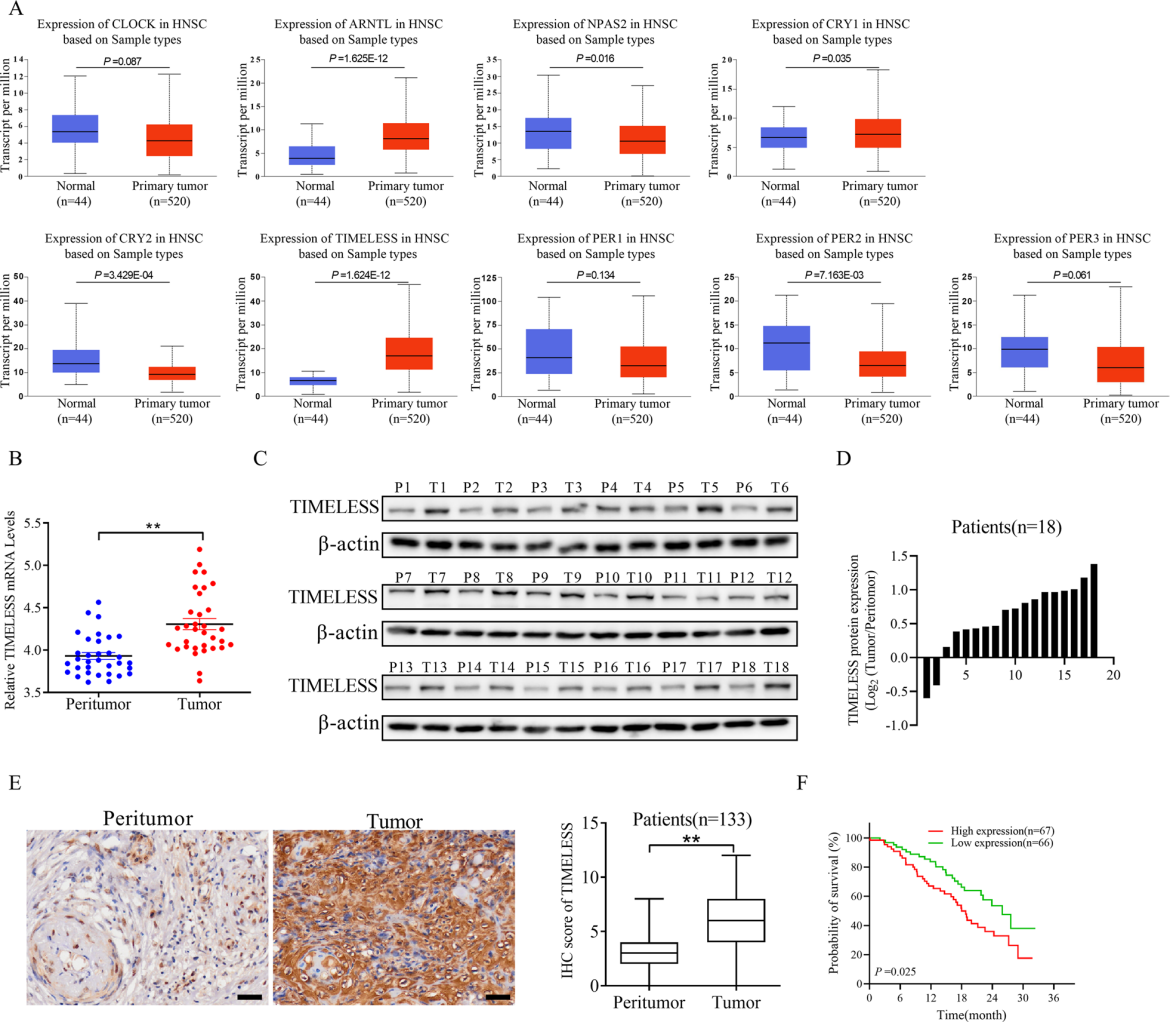
Upregulation of TIMELESS in OSCC and its correlation with poor prognosis. **A** The expression of core-clock genes in HNSC from TCGA database. **B** qRT-PCR analysis the expression of TIMELESS mRNA in 33 OSCC tissues. **C**, **D** Western blot analysis the expression of TIMELESS protein in 18 OSCC tissues. **E** IHC analysis the expression of TIMELESS in 133 OSCC tissues. Scale bar, 100 μm. *P < 0.05; **P < 0.01. **F** The prognostic analysis of TIMELESS expression was determined in 133 OSCC patients

Our initial investigation focused on the expression of core circadian genes in head and HNSC using data from the TCGA database. This analysis revealed a notable increase in the expression levels of *ARNTL, CRY1,* and *TIMELESS,* contrasted with decreased expression of *NPAS2* and *PER2* in HNSC. Notably, *TIMELESS* exhibited the most significant upregulation among these genes (*P* = 1.624E-12) (Fig. 1A). To further validate these findings, we examined some OSCC patient samples. Through qRT-PCR, Western blot, and IHC analyses, we observed a consistent pattern: *TIMELESS* expression was markedly higher in OSCC tissues than in adjacent non-cancerous tissues (Fig. 1B–E). Based on the aforementioned observations, the role of *TIMELESS* expression in prognosis of OSCC was investigated. *TIMELESS* expression is positively associated with clinical stage (*P* = 0.037), but not with other factors, including sex, age, and neoplasm histologic grade (Additional file 1: Table S1). The survival probability of patients with high *TIMELESS* expression was significantly lower than that of patients with low *TIMELESS* expression (*P* = 0.025) (Fig. 1F). This indicates a potential prognostic significance of *TIMELESS* for patients with OSCC.

### TIMELESS Enhances OSCC cell survival in vitro

**Fig. 2:**
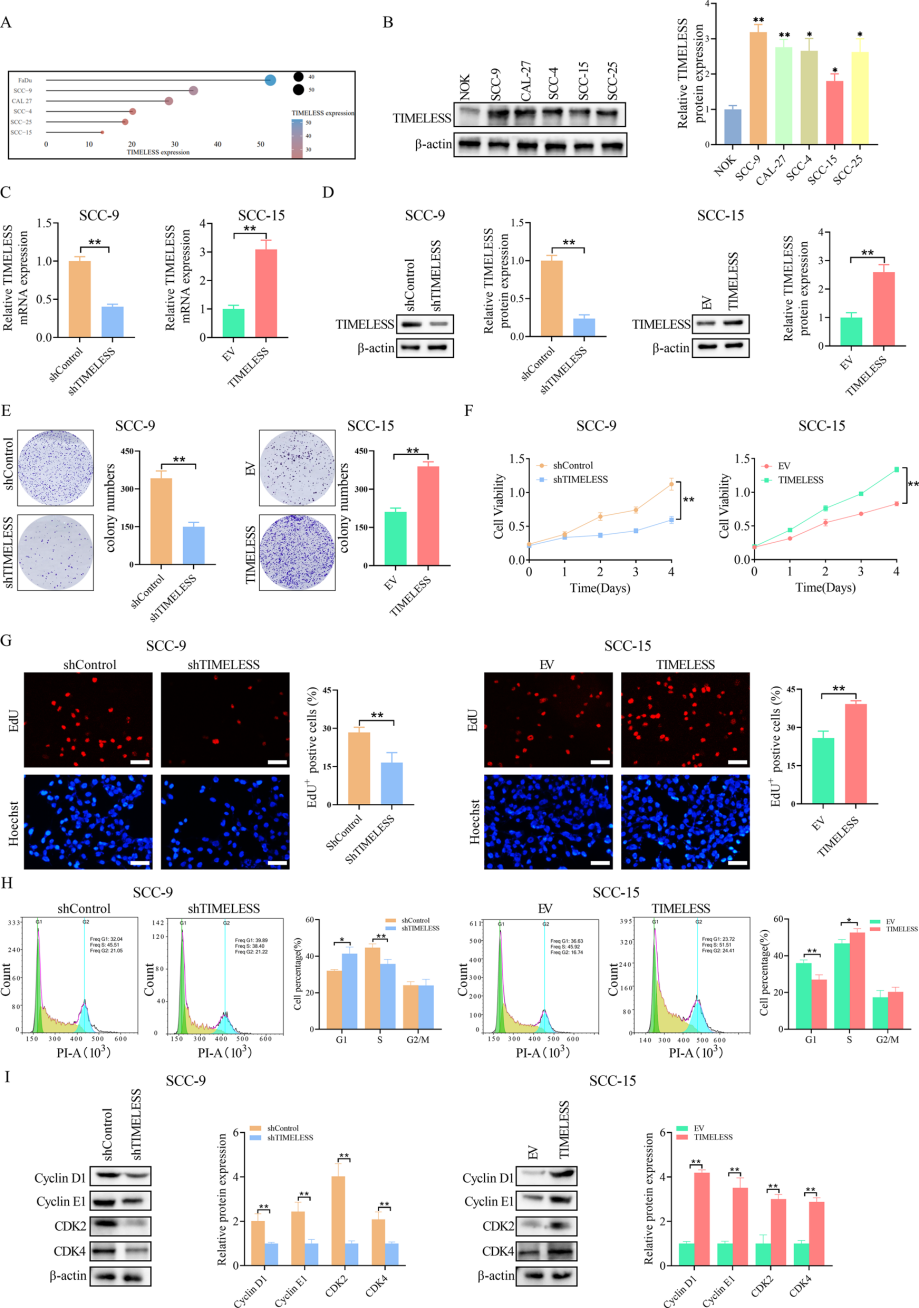
TIMELESS enhances OSCC cell survival in vitro. **A** TIMELESS mRNA expression in OSCC cell lines in CCLE database. **B** Expression level of TIMELESS in OSCC cell lines and a normal oral keratinocyte was analyzed by western blot. *P < 0.05; **P < 0.01. **C** The transfection effect was verified by qRT-PCR. **D** The transfection effect was verified by western blot. **E** Clonogenic colony formation assays. **F** The growth rates were analyzed by MTS assay. **G** Representative images of EdU-positive cells (EdU staining, red). Scale bar, 50 μm. **H** The statistics results of cell cycle analysis. Data shown were the mean ± S.E.M. from three independent experiments. **I** Cell cycle‐related proteins, including cyclin D1, cyclin E1, CDK2, and CDK4 were detected by western blotting

We embarked on examining the role of *TIMELESS* in OSCC both in vitro and in vivo. Initially, we assessed the mRNA expression of *TIMELESS* in various OSCC cell lines using the CCLE database, which revealed heightened expression levels (Fig. 2A). Subsequent Western blot analysis confirmed that *TIMELESS.*

protein expression was significantly higher in five OSCC cell lines (SCC-9, CAL-27, SCC-4, SCC-15, and SCC-25) compared to normal oral keratinocytes (NOK) (Fig. 2B). Based on these findings, SCC-15 cells, with lower native expression, were selected for overexpression studies, while SCC-9 cells, exhibiting higher *TIMELESS* expression, were used for knockdown experiments. The resulting stably transfected cell lines were validated using qRT-PCR and Western blot analysis (Fig. 2C and D).

To assess cell proliferation, we conducted colony formation, MTS, and EdU assays on OSCC cell lines. Our findings revealed that knocking down *TIMELESS* in SCC-9 cells led to a significant reduction in cell growth, as evidenced by diminished colony formation and a lower number of EdU positive cells, in comparison to control groups. Conversely, overexpressing *TIMELESS* in SCC-15 cells resulted in enhanced colony formation, increased cell proliferation, and a higher count of EdU positive cells (Fig. 2E, G). Moreover, we also have overexpressed *TIMELESS* in SCC-9 cells and knocked down *TIMELESS* in SCC-15 cells. The stable transfected cell lines were screened out successfully and verified by qRT-PCR and western blot (Additional file 1: Fig S1A and B). For detecting the cell proliferation ability, colony formation assay and MTS assay were performed. Above experimental results displayed that compared to the corresponding control groups, overexpression of *TIMELESS* promoted colony formation and cell growth ability in SCC-9 cells. Knockdown of *TIMELESS* decreased cell growth ability GLUT1and weakened colony formation in SCC-15 cells (Additional file 1: Fig S1C and D).

The influence of *TIMELESS* modulation was also evident in the cell cycle distribution. Specifically, *TIMELESS* knockdown in SCC-9 cells increased the proportion of cells in the G1 phase while decreasing those in the S phase. In contrast, overexpressing *TIMELESS* in SCC-15 cells showed an inverse effect, with a decreased G1 phase population and an increased S phase population (Fig. 2H). This shift in cell cycle phases was accompanied by corresponding changes in the expression of cell cycle-related proteins. In SCC-9 cells, *TIMELESS* knockdown led to decreased levels of Cyclin D1, Cyclin E1, CDK2, and CDK4. On the other hand, *TIMELESS* overexpression in SCC-15 cells upregulated these proteins (Fig. 2I). These results suggest that *TIMELESS* may play a role in accelerating cell division and facilitating the transition from the G1 to the S phase of the cell cycle. Additionally, we evaluated the effect of *TIMELESS* modulation on apoptosis in these cell lines. Interestingly, no significant changes were observed in the number of apoptotic cells, indicating that the influence of *TIMELESS* on OSCC cells primarily pertains to proliferation rather than apoptosis (Additional file 1: Fig S2).

### Inhibition of OSCC tumor growth by TIMELESS knockdown in vivo

**Fig. 3:**
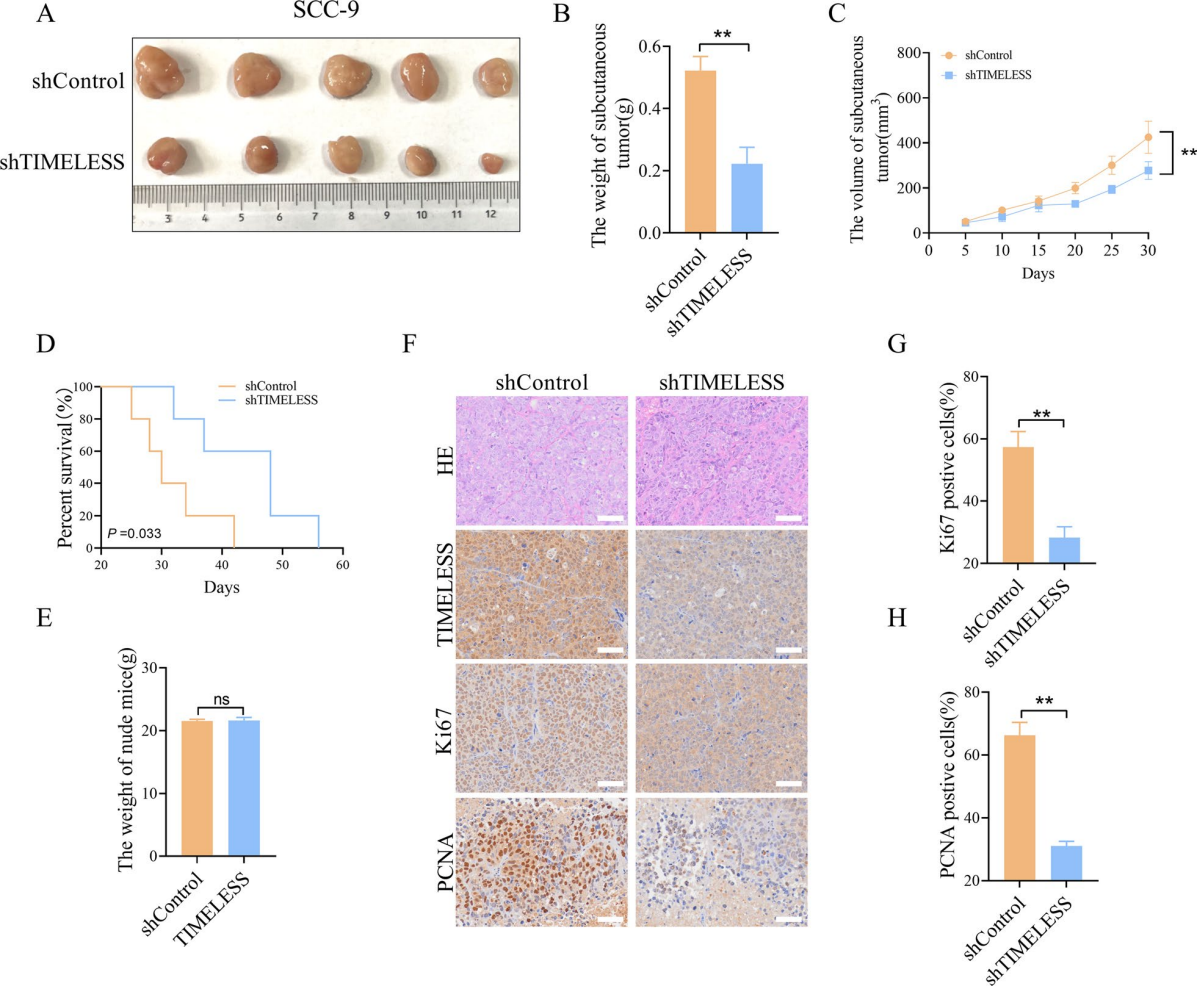
Inhibition of OSCC tumor growth by TIMELESS knockdown in vivo. **A**. The resected tumors from nude mice. **B** The weight of tumors in nude mice. *P < 0.05; **P < 0.01. **C** Tumor volume changes in nude mice. **D** Survival analysis on nude mice. **E** The body weight of nude mice. **F** HE staining, IHC staining of TIMELESS, Ki67 and PCNA in nude mice tumor tissues. Scale bar, 50 μm. **G** Comparison of Ki-67-positive cells in tumor tissues of nude mice xenograft model with different treatment as indicated. **H** Comparison of PCNA-positive cells in tumor tissues of nude mice xenograft model with different treatment as indicated

In vivo studies were conducted using a nude mouse model to assess the effects of *TIMELESS* knockdown on OSCC growth. We injected SCC-9 control cells and SCC-9 cells with stable *TIMELESS* knockdown subcutaneously into nude mice. This approach resulted in a 100% tumor formation rate in all 10 mice, with no instances of spontaneous mortality observed during the experiment. The growth of these tumor cells under the skin led to the formation of visible tumor masses (Fig. 3A). Comparative analysis between the control group and the *TIMELESS* knockdown group revealed a notable reduction in tumor size and weight in the latter, indicating that TIMELESS knockdown effectively inhibited tumor growth in the xenograft model (Fig. 3B-C). Additionally, the lifespan of the mice subjected to *TIMELESS* knockdown was significantly extended (*P* = 0.033) (Fig. 3D). It is noteworthy that there were no substantial differences in the overall body weight of the mice across the various treatment groups (Fig. 3E), suggesting the specificity of *TIMELESS* knockdown effects on tumor growth rather than general health. Histological examination of the xenografts further supported these findings. Tumors developed from SCC-9 cells with stable *TIMELESS* knockdown showed a marked decrease in Ki67 and PCNA staining, reflecting a lower cellular proliferation rate in these tumors (Fig. 3F-H). This decrease in proliferative markers confirms the critical role of TIMELESS in driving OSCC tumor growth in vivo.

### TIMELESS modulates glucose metabolism in OSCC cells

**Fig. 4:**
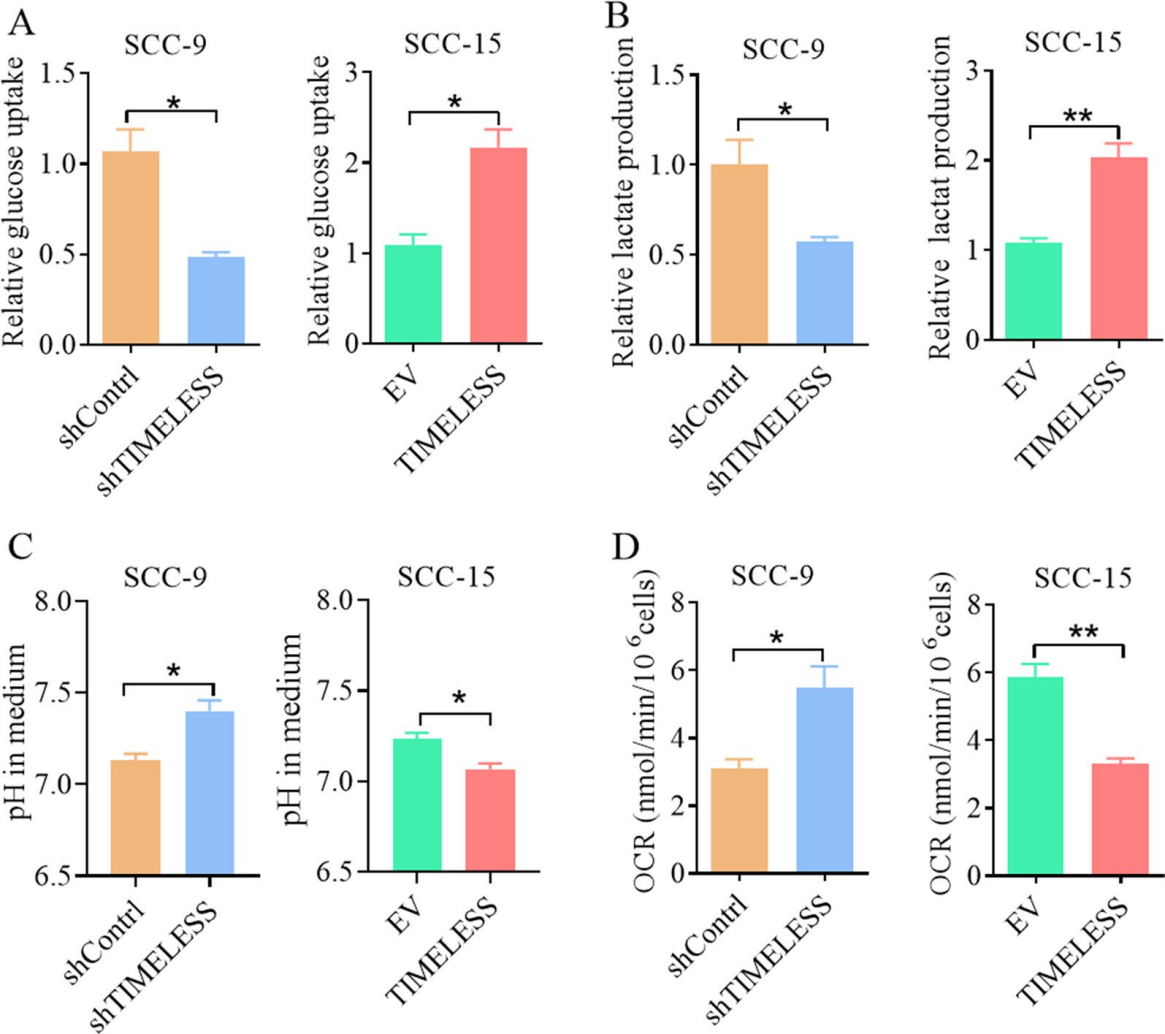
TIMELESS modulates glucose metabolism in OSCC cells. **A** The level of glucose uptake was examined. **B** Lactate production was examined. **C** Cell medium pH. **D** Oxygen consumption level of cell. Data shown were the mean ± S.E.M. from three independent experiments. *P < 0.05; **P < 0.01

In exploring the role of *TIMELESS* in cellular metabolism, we focused on its impact on glucose metabolism, a key aspect of tumor cell proliferation. We conducted glucose metabolic phenotype assays on stably transfected OSCC cells, measuring glucose uptake, lactate production, cell media pH, and oxygen consumption rate as per the manufacturer’s protocols. Our results indicated that *TIMELESS* knockdown in SCC-9 cells led to a decrease in both glucose uptake and lactate production. In contrast, overexpressing *TIMELESS* in SCC-15 cells enhanced these glycolytic processes (Fig. 4A, B). Additionally, *TIMELESS* knockdown was associated with an increase in oxygen consumption rate and pH in SCC-9 cells, suggesting a shift towards oxidative phosphorylation. Conversely, overexpressing *TIMELESS* in SCC-15 cells resulted in reduced oxygen consumption and lower pH levels, indicative of enhanced glycolysis (Fig. 4C, D). We also assessed the metabolic phenotype of fatty acids in OSCC cells but did not observe significant differences in the levels of free fatty acids, cholesterol, or phospholipids (Additional file 1: Fig S3). Further analysis revealed that overexpressing *TIMELESS* in SCC-9 cells promoted glucose uptake and lactate production, while its knockdown in SCC-15 cells had the opposite effect (Additional file 1: FigS4A, B). Similarly, *TIMELESS* overexpression in SCC-9 cells reduced oxygen consumption and pH, whereas its knockdown in SCC-15 cells led to increased oxygen consumption and pH (Additional file 1: FigS4C, D). These findings suggest that *TIMELESS* plays a significant role in promoting glycolysis and inhibiting mitochondrial oxidative phosphorylation in OSCC cells, thereby contributing to the metabolic reprogramming associated with tumor growth and survival.

### TIMELESS augments glycolysis via upregulation of key metabolic molecules

**Fig. 5:**
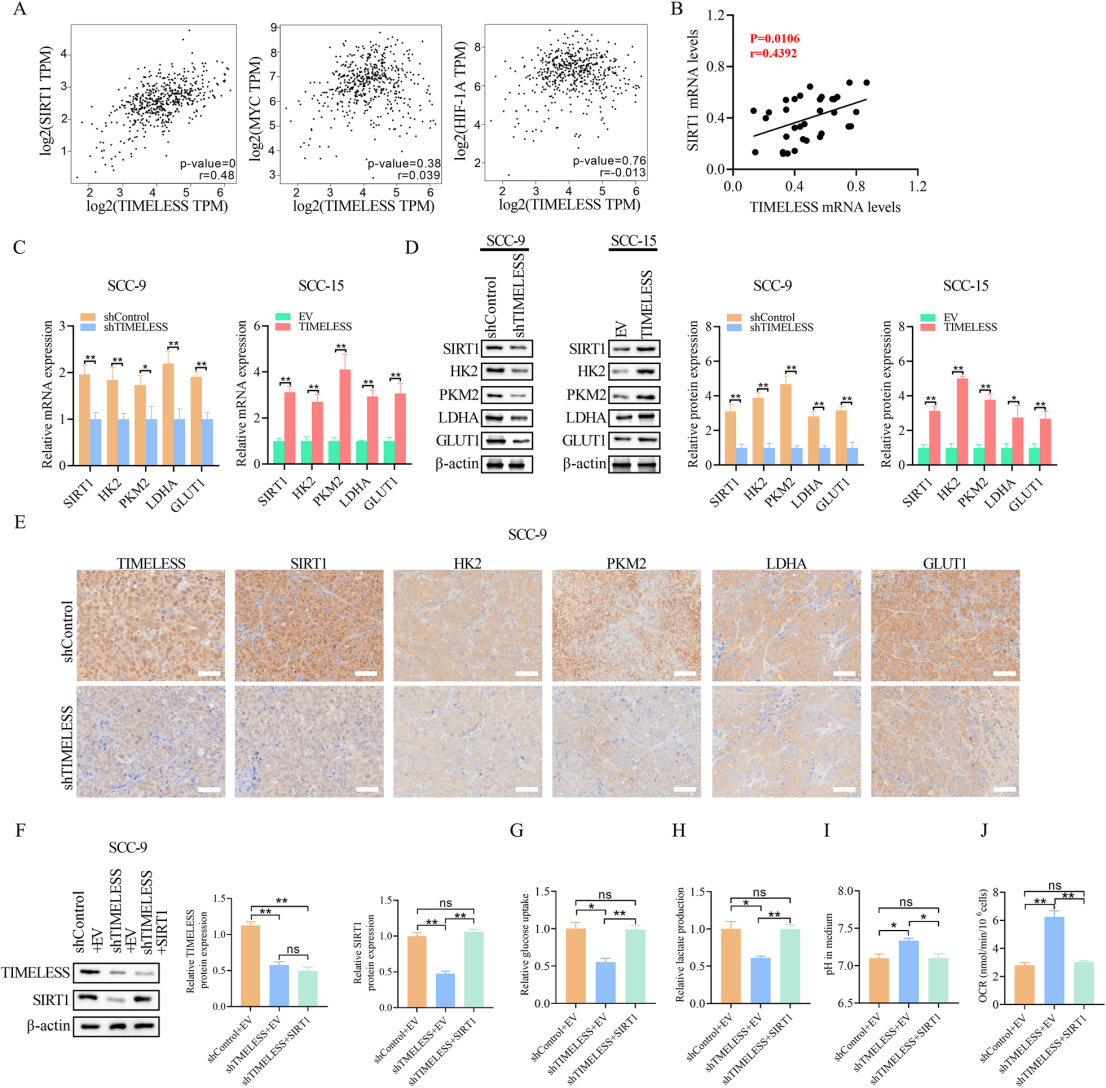
TIMELESS augments glycolysis via upregulation of key metabolic Molecules. **A** Correlation analysis between TIMELESS expression and SIRT1, HIF1A and MYC in HNSC tissues form TCGA database. **B** Correlation analysis between TIMELESS mRNA levels and SIRT1 mRNA levels in 33 OSCC tissues. **C** qRT-PCR analysis of the expression levels of TIMELESS and key glycolysis genes in OSCC cells. **D** Western blot analysis of the expression levels of TIMELESS and key glycolysis genes in OSCC cells. **E** IHC staining of TIMELESS, SIRT1, HK2, PKM2, LDHA and GLUT1 in nude mice tumors. **F** The transfection efficiency was examined with western blot. **G** The level of glucose uptake was examined. **H** Lactate production was examined. **I** Cell medium pH. **J** Oxygen consumption level of cell. Data shown were the mean ± S.E.M. from three independent experiments. *P < 0.05; **P < 0.01

Having established *TIMELESS*'s role in altering glucose metabolism in OSCC cells, we next sought to understand the underlying mechanisms. We analyzed the correlation between *TIMELESS* expression and key metabolic genes, including *SIRT1*, *HIF1A*, and *MYC*, using data from the TCGA HNSC database. This analysis revealed a significant positive correlation between *TIMELESS* and *SIRT1* levels (*P* = 0, r = 0.48) (Fig. 5A). Further analysis of OSCC tissues reinforced this correlation, showing a significant positive relationship between *TIMELESS* and *SIRT1* mRNA levels (*P* = 0.0106, r = 0.4392) (Fig. 5B). Western blot and qRT-PCR analyses further supported these findings, demonstrating that *SIRT1* expression increased with *TIMELESS* overexpression and decreased following its knockdown (Fig. 5C and D). Immunohistochemistry (IHC) in tumor tissues from nude mice also showed reduced *SIRT1* expression upon *TIMELESS* knockdown (Fig. 5E). These observations led us to hypothesize that *TIMELESS* might regulate glucose metabolism predominantly through *SIRT1* modulation.

To test this hypothesis, we overexpressed *SIRT1* in *TIMELESS* knockdown SCC-9 cells, confirming the transfection efficiency by Western blot (Fig. 5F). Results showed that *TIMELESS* knockdown decreased glucose uptake and lactate production in OSCC cells, which was reversed by *SIRT1* overexpression (Fig. 5G and H). Similarly, assays measuring oxygen consumption rate and pH indicated that *TIMELESS* knockdown increased these parameters in OSCC cells, whereas *SIRT1* overexpression attenuated these effects (Fig. 5I and J).

We further investigated whether *TIMELESS* influences the expression of glycolytic genes such as *HK2, PKM2, LDHA*, and GLUT1 through *SIRT1*. Our data showed that overexpression of *TIMELESS* significantly increased the expression of these glycolytic enzymes, which was reduced upon *TIMELESS* knockdown (Fig. 5C–E). These results collectively suggest that *TIMELESS* plays a crucial role in the regulation of glycolysis in OSCC cells, predominantly by modulating *SIRT1* and thereby influencing the expression of key glycolytic enzymes.

## Discussion

The significance of circadian genes in cancer development has increasingly come into focus in recent years. A body of evidence highlights that the circadian gene *TIMELESS* is notably upregulated in various cancers, *including breast*, cervical, nasopharyngeal, and prostate cancers, where its elevated expression is linked to enhanced tumor growth [16–20]. Our study aligns with these findings, revealing a marked increase in *TIMELESS* expression in OSCC tissues and cells, which correlates with a poorer prognosis. Additionally, our results extend this understanding by demonstrating that *TIMELESS* not only promotes OSCC cell proliferation but also contributes to tumor growth both in vitro and in vivo. These outcomes reinforce the potential of *TIMELESS* as a prognostic marker in OSCC. While the connection between circadian rhythm disruption and cancer genesis is well-documented, the specific role of *TIMELESS* in the metabolic reprogramming of OSCC cells has not been thoroughly explored. Metabolic reprogramming, particularly the shift towards glycolysis, is a defining characteristic of tumor cells and plays a crucial role in cancer development and progression. Our research sheds light on this aspect by illustrating how *TIMELESS* influences glucose metabolism in OSCC, promoting glycolytic processes, and inhibiting oxidative phosphorylation. This insight into *TIMELESS*'s role in metabolic alteration provides a deeper understanding of its contribution to OSCC pathogenesis and highlights the gene's potential as a target for therapeutic interventions.

Core circadian genes are integral in regulating a wide array of metabolic processes, including glucose and lipid metabolism, oxidative phosphorylation, and mitochondrial dynamics [25]. Disruptions in these circadian mechanisms have been implicated in metabolic disorders such as hyperlipidemia. For instance, *BMAL1* regulates the synthesis of primordial lipoproteins and their expansion into larger forms by controlling MTP and ApoAIV through transcription factors Shp and Crebh [26]. Similarly, altered transcriptional cycling of core clock genes like *BMAL1, CLOCK,* and *PER3* in skeletal muscle is associated with disrupted circadian rhythms in type 2 diabetese [27]. Furthermore, REV-ERB nuclear receptors, crucial components of the molecular clock, play a significant role in controlling the circadian period and modulating metabolic responses, such as diet-induced obesity [28]. One of the hallmark features of malignant tumors is the reprogramming of energy metabolism. Typically, eukaryotic cells utilize mitochondrial oxidative phosphorylation to fully oxidize glucose, their primary energy source, in the presence of oxygen [29]. However, cancer cells often diverge from this pathway, preferring aerobic glycolysis even under sufficient oxygen levels, a phenomenon known as the Warburg effect [30]. The circadian system exerts a profound influence on glucose metabolism and regulates a vast array of physiological and metabolic functions in mammals [31]. The extent to which circadian genes modulate metabolic reprogramming in OSCC is not fully understood. Our study reveals that in OSCC cells, the overexpression of *TIMELESS* increases glycolytic capacity while reducing oxidative phosphorylation, with *TIMELESS* knockdown cells exhibiting the opposite trend. This shift toward aerobic glycolysis, which is characterized by increased lactate production and reduced pH in the tumor microenvironment, aligns with the activation of glycolytic genes. We observed that the expression of *SIRT1, HK2, PKM2, LDHA,* and GLUT1 is upregulated with increased *TIMELESS* expression and downregulated upon its knockdown. These findings indicate that *TIMELESS* plays a pivotal role in promoting the glycolytic phenotype in OSCC cells, likely through the regulation of *SIRT1* and key glycolytic enzymes, thereby contributing to the progression of OSCC.

Silent information regulator 1 (*SIRT1*), a NAD + -dependent histone deacetylase, plays a crucial role in various cellular processes, including gene silencing, cell cycle regulation, fat and glucose metabolism, oxidative stress response, and cellular senescence [32, 33]. It is known for deacetylating the tumor suppressor protein p53, which is its first identified non-histone substrate. This action of *SIRT1* on p53 results in the modulation of cellular metabolism, particularly by enhancing mitochondrial oxidative phosphorylation and suppressing aerobic glycolysis [34]. Research in pancreatic cancer has demonstrated that a loss of *SIRT1* correlates with reduced expression of glycolytic pathway proteins like GLUT1 and decreased cancer cell proliferation [35]. Conversely, *SIRT1* overexpression has been found to upregulate GLUT1 transcription and promote both cell proliferation and glycolysis in bladder cancer cells [36]. Additionally, the AMPK/SIRT1 pathway significantly influences glycolysis regulation in response to follicle-stimulating hormone in follicular granulosa cells, with *SIRT1* activation leading to increased glycolytic protein expression and lactic acid production [37]. Given *SIRT1*'s pivotal role as a metabolic sensor, we investigated its interaction with *TIMELESS* in the context of OSCC. Our analysis indicates a positive correlation between the expression of *TIMELESS* and *SIRT1* in head and HNSC. We found that knocking down *TIMELESS* reduces *SIRT1* activity, thereby promoting glycolysis and inhibiting oxidative phosphorylation. Conversely, overexpression of *SIRT1* reverses these metabolic effects. Notably, changes in *TIMELESS* expression were paralleled by similar trends in the expression of downstream glycolytic targets such as *HK2, PKM2, LDHA*, and GLUT1 in OSCC cells. These findings lead us to propose that *TIMELESS* contributes to OSCC progression by enhancing aerobic glycolysis. This effect is likely mediated through *SIRT1*, which, in turn, regulates the expression of key glycolytic enzymes and GLUT1.

Current research increasingly links circadian gene dysregulation to cancer development. Our study reveals that in OSCC, *TIMELESS* is overexpressed, leading to suppressed oxidative phosphorylation and increased glycolysis and cell proliferation. Notably, knocking down *TIMELESS* and subsequently overexpressing *SIRT1* in OSCC cells reversed the glycolytic changes observed with *TIMELESS* knockdown alone. These findings suggest that *TIMELESS*'s tumor-promoting role is mediated through *SIRT1* activation and glycolysis regulation, positioning *TIMELESS* as a potential therapeutic target for addressing metabolic anomalies in OSCC.

Our study offers novel insights into the critical regulatory role of *TIMELESS* in the glucose metabolism and progression of OSCC. These findings enhance our understanding of the biological mechanisms driving OSCC progression. Given its influence on tumor growth, *TIMELESS* emerges as a promising biomarker for OSCC diagnosis and a potential therapeutic target.

## Conclusions

TIMELESS facilitates OSCC cell growth primarily by enhancing glycolysis and suppressing oxidative phosphorylation, a process mediated through the activation of SIRT1.

### Supplementary Information

**Additional file 1: Figure S1:** TIMELESS promotes OSCC cell growth in vitro. (A) The transfection effect was verified by qRT-PCR. (B) The transfection effect was verified by western blot. (C) Clonogenic colony formation assays. (D) The growth rates were analyzed by MTS assay. Data shown were the mean ± S.E.M. from three independent experiments. *P<0.05; **P<0.01.** Figure S2. **Apoptosis detection in cell transfection models. Data shown were the mean ± S.E.M. from three independent experiments. **Figure S3.** Analysis the ability of fatty-acid oxidation of OSCC cells (A) Intracellular level of free fatty acid was determined in OSCC cells. (B) Intracellular level of cholesterol was determined in OSCC cells. (C) Intracellular level of phospholipid was determined in OSCC cells. Data shown were the mean ± S.E.M. from three independent experiments. **Figure S4. **TIMELESS promotes glycolysis and inhibits oxidative phosphorylation in OSCC cells. (A) The level of glucose uptake was examined. (B) Lactate production was examined. (C) Cell medium pH. (D) Oxygen consumption level of cell. Data shown were the mean ± S.E.M. from three independent experiments. *P<0.05; **P<0.01. **Table S1.** Clinical characteristics of 133 patients and the expression of TIMELESS in OSCC tissues.

## Data Availability

The data applied in the bioinformatics analysis were obtained from TCGA and CCLE open-access database. The datasets used and/or analyzed during the current study are available from the corresponding author on reasonable request.

## Abbreviations

OSCC: Oral squamous cell carcinoma
HNSC: Head and neck squamous cell carcinoma
qRT-PCR: Quantitative real-time PCR
IHC: Immunohistochemistry
CCLE: Cancer Cell Line Encyclopedia database
TCGA: The Cancer Genome Atlas
SIRT1: Silent information regulator 1
HK2: Hexokinase2
PKM2: Pyruvate kinase isozyme typeM2
GLUT1: Glucose transporter 1
LDHA: Lactate Dehydrogenase A
