# Supplementary material for: TIMELESS promotes reprogramming of glucose metabolism in oral squamous cell carcinoma
Source: J Transl Med. 2024 Jan 4;22:21. doi: 10.1186/s12967-023-04791-3 (PMC10768318; doi:10.1186/s12967-023-04791-3)
Supplement: Supplementary file 1 — Additional file 1: Figure S1: TIMELESS promotes OSCC cell growth in vitro. (A) The transfection effect was verified by qRT-PCR. (B) The transfection effect was verified by western blot. (C) Clonogenic colony formation assays. (D) The growth rates were analyzed by MTS assay. Data shown were the mean ± S.E.M. from three independent experiments. *P<0.05; **P<0.01. Figure S2. Apoptosis detection in cell transfection models. Data shown were the mean ± S.E.M. from three independent experiments. Figure S3. Analysis the ability of fatty-acid oxidation of OSCC cells (A) Intracellular level of free fatty acid was determined in OSCC cells. (B) Intracellular level of cholesterol was determined in OSCC cells. (C) Intracellular level of phospholipid was determined in OSCC cells. Data shown were the mean ± S.E.M. from three independent experiments. Figure S4. TIMELESS promotes glycolysis and inhibits oxidative phosphorylation in OSCC cells. (A) The level of glucose uptake was examined. (B) Lactate production was examined. (C) Cell medium pH. (D) Oxygen consumption level of cell. Data shown were the mean ± S.E.M. from three independent experiments. *P<0.05; **P<0.01. Table S1. Clinical characteristics of 133 patients and the expression of TIMELESS in OSCC tissues. [file 12967_2023_4791_MOESM1_ESM.docx]

**Additional file**

**TIMELESS promotes reprogramming of glucose metabolism in oral squamous cell carcinoma**

**Additional file Figures**


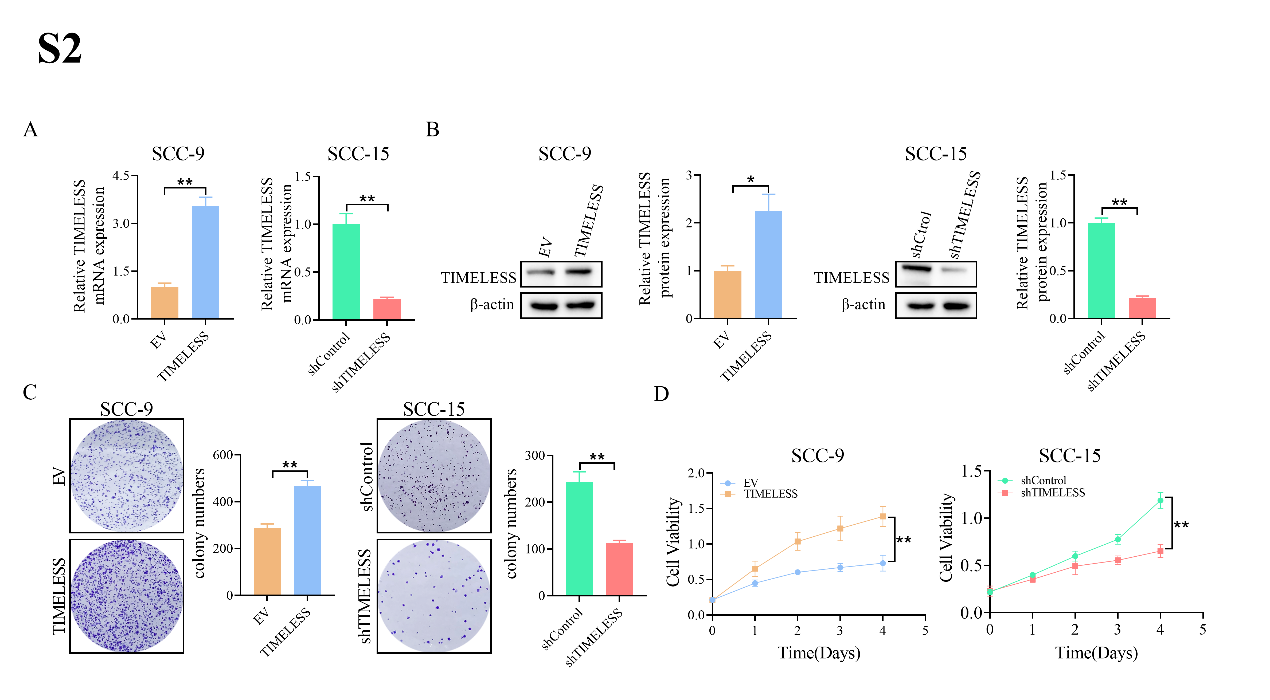
**Figure S1:** TIMELESS promotes OSCC cell growth in vitro.

(A) The transfection effect was verified by qRT-PCR.

(B) The transfection effect was verified by western blot.

(C) Clonogenic colony formation assays.

(D) The growth rates were analyzed by MTS assay. Data shown were the mean ± S.E.M. from three independent experiments. **P*<0.05; ***P*<0.01.


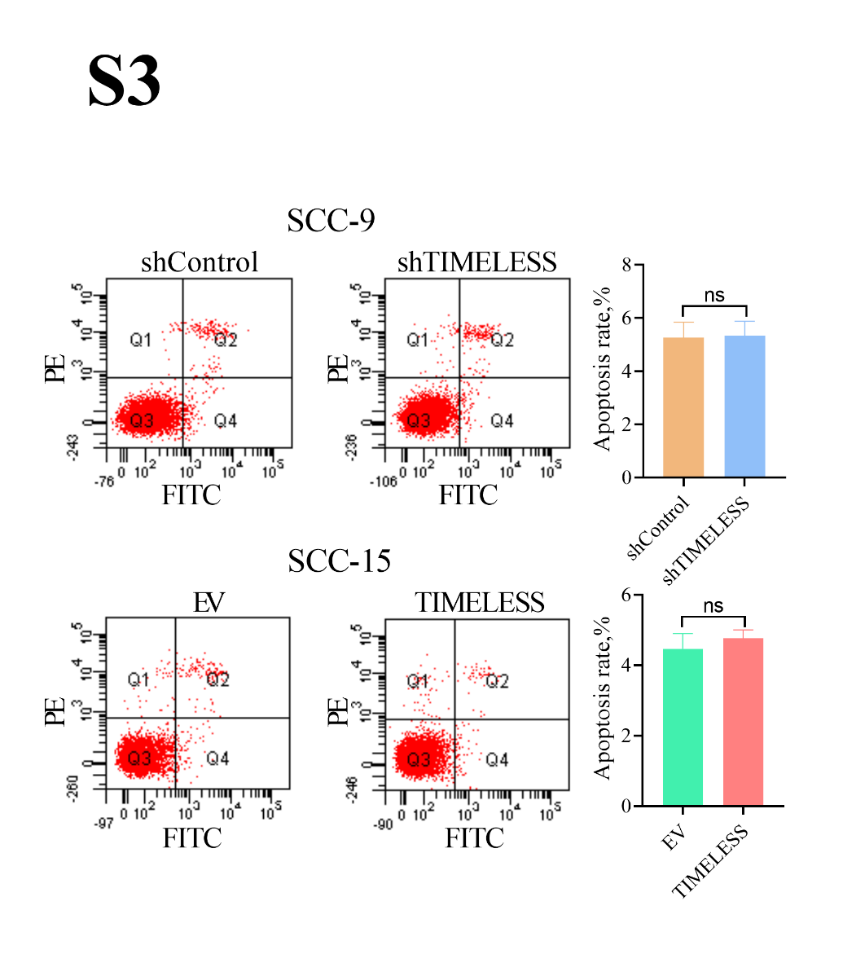


**Figure S2.** Apoptosis detection in cell transfection models. Data shown were the mean ± S.E.M. from three independent experiments.


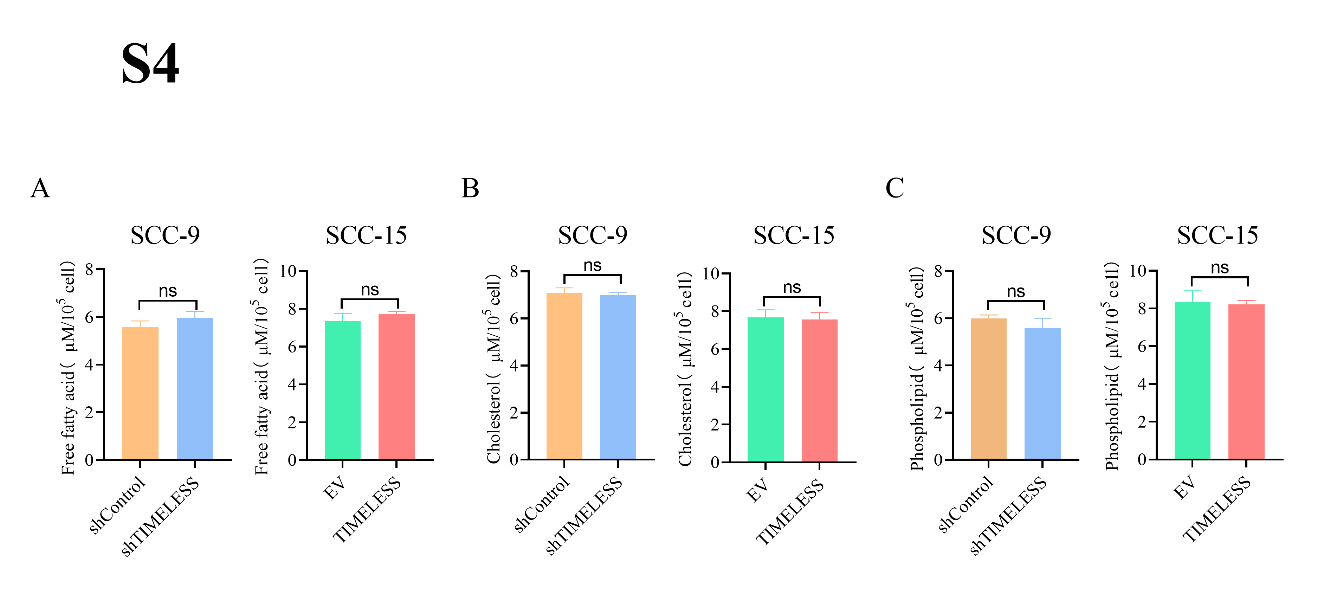


**Figure S3.** Analysis the ability of fatty-acid oxidation of OSCC cells

(A) Intracellular level of free fatty acid was determined in OSCC cells.

(B) Intracellular level of cholesterol was determined in OSCC cells.

(C) Intracellular level of phospholipid was determined in OSCC cells. Data shown were the mean ± S.E.M. from three independent experiments.


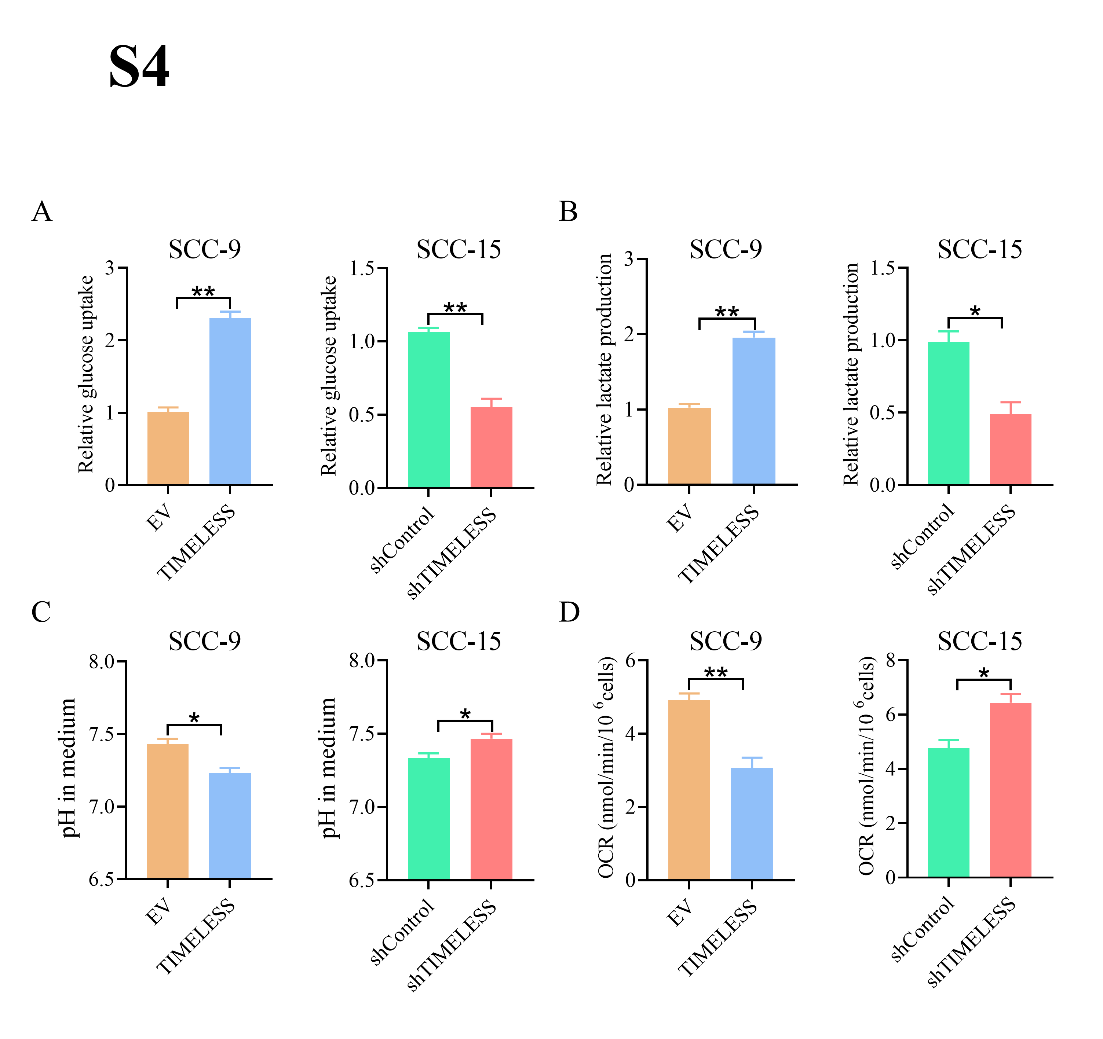


**Figure S4.** TIMELESS promotes glycolysis and inhibits oxidative phosphorylation in OSCC cells.

(A) The level of glucose uptake was examined.

(B) Lactate production was examined.

(C) Cell medium pH.

(D) Oxygen consumption level of cell. Data shown were the mean ± S.E.M. from three independent experiments. **P*<0.05; ***P*<0.01.

**Additional file Table S1. Clinical characteristics of 133 patients and the expression of TIMELESS in OSCC tissues.**

| Variables | No. of cases | TIMELESS expression | | *P* value |
| --- | --- | --- | --- | --- |
|  |  | Low | High |  |
| All | 133 | 66 | 67 |  |
| Age |  |  |  | 0.852 |
| <55 | 41 | 21 | 20 |  |
| >=55 | 92 | 45 | 47 |  |
| Gender |  |  |  | 0.447 |
| Female | 38 | 21 | 17 |  |
| Male | 95 | 45 | 50 |  |
| Clinical stage  Ⅰ—Ⅱ | 32 | 25 | 14 | 0.037 |
| Ⅲ—Ⅳ | 101 | 41 | 53 |  |
| neoplasm histologic grade |  |  |  | 0.072 |
| G1-2 | 87 | 47 | 40 |  |
| G3-4 | 46 | 19 | 27 | ` |

*P* value < 0.05 was considered statistically significant.
